# Professor Cuifen Huang: a great molecular geneticist and the founder of genetic engineering in China

**DOI:** 10.1007/s13238-019-0620-5

**Published:** 2019-04-03

**Authors:** Wei Gong, Leyi Cui, Yike Ying, Yijing Shen, Jiaqi Bao

**Affiliations:** Zhejiang Normal University, Jinhua, 321004 China

**Figure 1:**
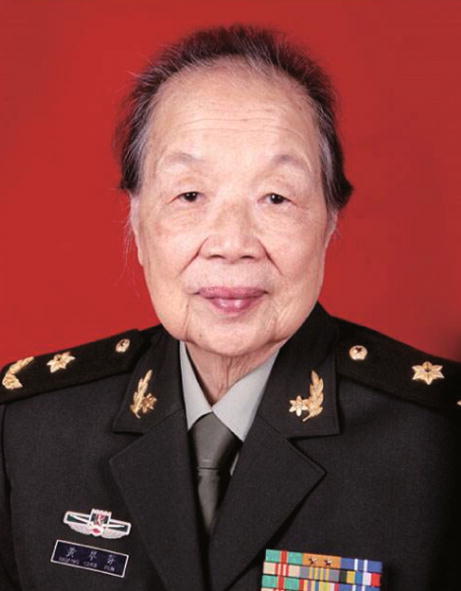
Prof. Huang attended the National Model Conference, 1987

Professor Cuifen Huang (黄翠芬, 1921–2011) is a famous molecular geneticist and one of the founders of Genetic Engineering in China (Fig. 1). She devoted all her life to researches on genetic engineering of vaccines and polypeptide drugs and studies on molecular tumors. She made tremendous contributions to the development of biotechnology and military medical science in China. She was also the first scientist who cloned and expressed the pro-urokinase gene in China which showed high selective thrombolysis without systemic hemorrhage (Chinese Academy of Engineering, 2009).

Prof. Huang was born in Guangdong, China in 1921. After receiving her bachelor’s degree in chemistry in Lingnan University (now known as Sun Yat-sen University), she went to the Microorganism Epidemiology Institute of Central Health Laboratory to work on the preparation of Penicillin, the extraction of antigens and the drug sensitivity test of Yersinia pestis. In 1948, she studied in Cornell University in USA for her master’s degree and was mentored by Professor J. Sherman who was also the editor-in-chief of the journal *Bacteriology*. Prof. Huang went back to China in 1950, shortly after the founding of People’s Republic of China. At the beginning, she was appointed as an associate professor in Shandong Medical College (now known as Shandong University School of Medicine). There, she was responsible for the compilation and teaching of Microbiology, and also performed researches on Bacteriology. In 1954, Prof. Huang started her researches on the toxicological mechanism of microbial toxins and the integrated control methods of epidemics in the Academy of Military Medical Sciences. After years of hard work, she successfully synthesized Quadrivalent Traumatic toxoid for the first time. In 1980, Prof. Huang established the first molecular genetics laboratory of People’s Liberation Army of China (PLA), and focused on the researches on hepatitis B core antigen. In the mid-1980s, Prof. Huang moved to the Institute of Bioengineering and studied in the field of Genetic Engineering. In the late 1990s, Prof. Huang started her researches on molecular tumors.

Prof. Huang accomplished remarkable achievements in genetic engineering, such as her researches on hepatitis B core antigen and e antigen, diarrhea vaccines, cholera toxin, thrombolytic agents. Given Prof. Huang’s outstanding achievements, she was honored the First Class Prize for Scientific and Technology Progress (STP) of China in 1987 and 1995, and obtained a number of Military Prizes for STP of PLA. In October 1984, Prof. Huang was awarded the national honorary title of “Advanced worker in Scientific and Technological research” by Deng Xiaoping, the supreme leader of China at that time. In 1996, Prof. Huang was elected as an academician of Chinese Academy of Engineering. In addition, Prof. Huang was invited for several times by the leaders of China to attend the National Ceremony at Tiananmen Square as an outstanding representative of the scientific and technological community.

Prof. Huang participated in multiple major national projects such as National Key Technology Research and Development Program of China during the “7th Five-Year Plan” and National High Technology Research and Development Program of China (National 863 Program), and genetic engineering was her top priority. Prof. Huang was the leading scientist of the National 863 Program—genetic engineering vaccine for human bacterial diarrhea. During this program, Prof. Huang studied the essential pathogen, enterotoxigenic *Escherichia coli* (ETEC), which caused human diarrhea. ETEC contain two types of noxious-gene: heat-labile toxin (LT) and heat-stable toxin (ST). Prof. Huang and her team used genetic engineering technology to fuse the B-subunits of LT (LTB) together with ST to obtain LTB/pro-ST fusion protein, which not only retained the immunity of LTB, but also endowed ST with immunity and reduced the biological toxicity. Prof. Huang also published several research articles to present these research data, such as “Fusion of genes encoding *Escherichia coli* heat-labile and heat-stable enterotoxins” (Zhang et al., 2000), “Research progress in the prevention of bacterial diarrhea vaccine by genetic engineering technology” (Huang, 1986), which laid a solid foundation for the production of humanized vaccines in the future. Another outstanding study is the cloning and expressing of pro-urokinase, which was supervised by Prof. Huang since 1982. She added a signal peptide containing 20 amino-acids to pro-urokinase that has 411 amino-acids using gene cloning, cloned and expressed the pro-urokinase for the first time in China (Fang et al., 1990). At the same time, she modified the structure of human tissue plasminogen activator and obtained one kind of anti-thrombolytic polypeptide drug (Zhou et al., 1998). All these research findings reached the leading levels in the world at that time.

Prof. Huang devoted her entire life to the scientific researches in China. Due to the confidential policy of national projects, she gave up the right to publish many research findings. Even though, Prof. Huang made outstanding achievements with her research team and published dozens of high quality research articles in multiple academic journals. Meanwhile, she edited several monographs including *Advances in Molecular Biology of Medical Bacteria* and *Theory and Methods of Genetic Engineering*, compiled the textbook *Bacteriology*, which comprehensively introduced the development and evolution of bacteria and was one of the first textbooks in the field. In addition, she coauthored *Pharmacology* with her husband, Professor Tingchong Zhou (周廷冲) (General Administration Department, 1990).

Prof. Huang was also a great mentor in the cultivation of young talents. After returning to China in 1950, Prof. Huang insisted on teaching by words and deeds, she encouraged young scholars to express their opinions in public places, not giving ways to so-called academic authority. Prof. Huang has mentored and trained a large number of outstanding talents in the field of biotechnology, such as Peitang Huang (黄培堂) and Xiao Yang (杨晓). Peitang Huang is the chief scientist of the Major State Basic Research Development Program of China (National 973 Program) and vice chairman of the scientific and technological research group of the National Severe Acute Respiratory Syndrome (SARS) Prevention and Control Center. He has published more than 50 articles in academic journals, and has won military prizes for STP of PLA for several times. Xiao Yang is the head of the transgenic group in China. In 1996, Prof. Huang recommended her graduate student Xiao Yang to go to USA to study gene knockout technology, who then published many high quality research articles and was recognized as the first person to master gene knockout technology in China.

**Figure 2:**
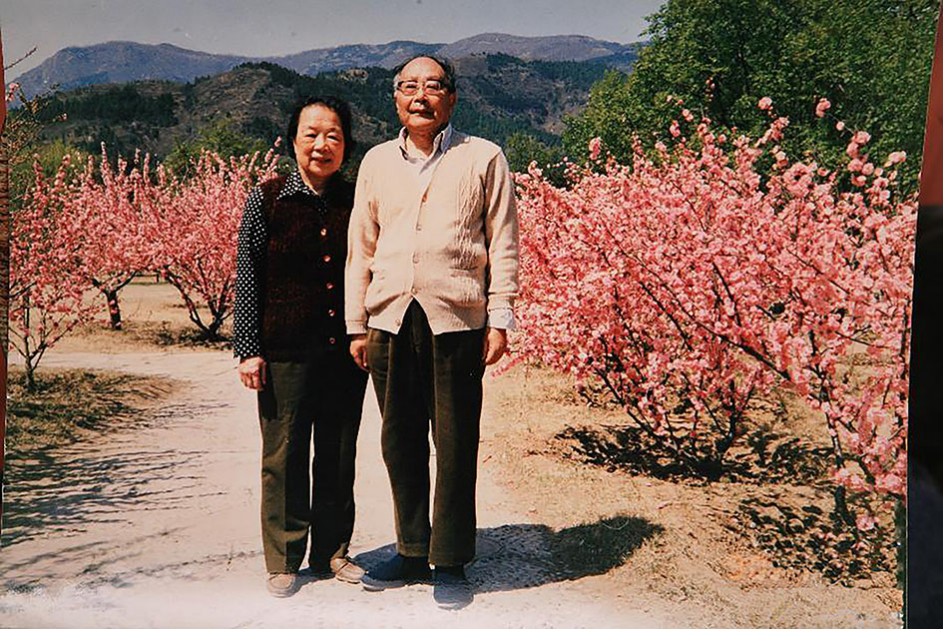
Prof. Huang and Prof. Zhou at the Zhongshan Park, 1995

Prof. Huang and her husband, Prof. Zhou were both academicians in the Military Medical College (Hao, 2013). They have been together for more than 40 years, conquered many difficulties together and made tremendous contributions to the development of science and technology in China. In August 1950, they overcame many difficulties and ventured into a cargo ship. After 56 days of wandering at sea, they finally returned to China (Hu et al., 1996). In 1977, when Prof. Zhou was severely sick at home, Prof. Huang was busy taking care of him. Meanwhile, she was still engaged in her researches. Later on, Prof. Huang’s meticulous care brought Prof. Zhou back to work, and they continued to work together for the development of science and technology in China. When they got some time for their personal life, Prof. Huang would cook for Prof. Zhou and they would take a walk in the park together (Fig. 2).

Prof. Huang passed away in Beijing in August 2011. She was a great geneticist and made outstanding contributions to the development of science and technology in China in terms of academic researches and cultivation of young talents, especially in the fields of genetic engineering of vaccines and polypeptide drugs and studies on molecular tumors. “Straight waist to be a ladder, bending over to be a bridge for others (直腰作人梯,弯腰为人桥)” is the true reflection of Prof. Huang’s life. Her down-to-earth research style, collaborating research personality and selfless research spirit are worthy of appreciation and learning by scientific researchers.
